# The Role of Leukotriene Receptor Signaling in Inflammation and Cancer

**DOI:** 10.1100/tsw.2007.200

**Published:** 2007-09-01

**Authors:** Ramin Massoumi, Anita Sjölander

**Affiliations:** Cell and Experimental Pathology, Department of Laboratory Medicine, Lund University, Malmo University Hospital, SE-205 02 Malmo, Sweden

**Keywords:** leukotrienesl, CysLT_1_ receptor, proliferation, surviva

## Abstract

Leukotrienes (LTs) and prostaglandins (PGs) are metabolites of arachidonic acid that play major roles in various inflammatory conditions. The release of these mediators, by cells recruited to or present at the site of inflammation, modulate/influence the magnitude of the inflammatory response. A better understanding of eicosanoids and how their receptors trigger intracellular signaling during inflammatory conditions is helping to elucidate the well-known connection between chronic inflammatory disease and neoplastic transformation. In the current review, we summarize the role of LTs and PGs in chronic inflammation and, in particular, we focus on recent insights into the role of CysLT_1_ receptor signaling pathway. In addition, we delineate how continuous CysLT_1_ receptor activation and signaling can increase cell survival and proliferation as important early steps toward oncogenicity.

## LEUKOTRIENES IN CHRONIC INFLAMMATION

The eicosanoids known as leukotrienes (LTs) and prostaglandins (PGs) are inflammatory mediators with potent biological activities in the pathogenesis of many diseases. In most of the chronic inflammatory conditions, such as psoriasis[1], rheumatoid arthritis[2], and inflammatory bowel disease (IBD)[3,4,5], the level of LTs and PGs are increased.

The strongest association of chronic inflammation with malignant diseases is seen in colon carcinogenesis arising in individuals with IBDs. Indeed, patients suffering from IBDs have about a 30-fold increased risk of developing colon cancer[6]. The role of eicosanoids is particularly relevant for two distinct IBD diseases: ulcerative colitis and Crohn's disease. In these chronic IBDs, elevated level of LTs and PGs increase the risk for development of cancer and, thereby, a reduced survival of these patients. As expected, it has been established that a cause-and-effect link between chronic inflammation and colon cancer occurs via activation and overexpression of the two enzymes, 5-lipoxygenase (5-LO) and cyclooxygenase (COX), responsible for regulating the production of LTs and PGs, respectively.

The expression of the COX-2 isoform, in contrast to COX-1, is affected by various stimuli and is highly up-regulated, both during inflammatory conditions and cancer diseases. For instance, COX-2 protein was detected in most IBDs and colon cancer tissues, but absent in adjacent normal tissue from the same colon. Furthermore, COX-2 mRNA levels were markedly increased in most of the human colorectal carcinomas, compared with paired samples of normal mucosa. The elevated PG production at the site of the tumor is a good indicator of increased COX-2 activity in colon cancer tissue[7,8,9]. Therefore, these studies support the concept that COX-2 and its product, $PGE_2$, levels are increased in IBD and colorectal tumors, and that specific inhibition of COX-2 appears to be a plausible approach to cancer prevention. There are vast amounts of data suggesting that nonsteroidal anti-inflammatory drugs (NSAIDs), such as NS-398, an inhibitor of COX-2, prevents the risk of colon cancer[10]. NS-398 was shown to suppress the proliferation of intestinal cancer cell lines that express high levels of COX-2[11], or COX-2 regulated colon carcinoma–induced angiogenesis[12]. Rofecoxib (Vioxx), a selective COX-2 inhibitor, prevented the recurrence of colorectal polyps among patients with a history of colorectal adenocarcinomas. Recently, celecoxib, another selective COX-2 inhibitor, received approval for cancer prevention in patients with familial adenomatous polyposis (FAP). However, Vioxx was withdrawn from the market in 2004 just before a 3-year clinical trail of adenomatous polyp prevention was ended because it was found to significantly increase the risk of heart disease.

The production of different LTs from arachidonic acid is dependent on the expression of 5-LO, an enzyme that regulates the first step in the synthesis of LTs. LTs are well-known mediators of acute inflammatory and immediate hypersensitivity responses. Induction of experimental colitis in mice lacking the 5-LO protein significantly reduced the degree of infiltration of inflammatory cells and colonic injury. This former effect was due to a reduced expression of adhesion molecules, such as P-selectin, E-selectin, ICAM-1, and VCAM-1, known to be responsible for the interaction between neutrophils and endothelial cells[13,14]. In contrast, no increased expression of 5-LO-mRNA has been detected in colonic biopsies from different IBD patients, regardless of their disease being in either a quiescent or active stage[15]. Even though the exact role of 5-LO during inflammation and carcinogenesis is still quite unclear, there is evidence that they are essential for regulation of apoptosis. A number of studies have shown that inhibition of 5-LO decreases growth and promotes cell death in several transformed and nontransformed cell lines[16,17,18].

In a recent tissue array study using colorectal cancer and control specimens, we found elevated levels of 5-LO and COX-2 in colorectal carcinomas. In accordance, similar observations were made in different colon carcinoma cell lines when these were compared to nontransformed intestinal epithelial cell lines[19]. Interestingly, we found that activation of $CysLT_1$ receptor signaling led to an increased COX-2-mediated production of $PGE_2$. This effect was accompanied by a translocation of COX-2 to the nuclear membrane. The activation of COX-2 was shown to be important for the survival of the intestinal epithelial cells[20]. These results suggest that $LTD_4$ mediated COX-2 activation and $PGE_2$ production have a major impact on intestinal epithelial cell survival.

## ROLE OF LEUKOTRIENE RECEPTORS DURING INFLAMMATION

The most potent LTs, $LTB_4$ and cysteinyl leukotrienes (CysLTs) $LTC_4$ and $LTD_4$, are synthesized primarily by stimulated leukocytes and, to a lesser extent, by other types of cells, including epithelial cells and endothelial cells at the site of inflammation[21]. Two receptors for $LTB_4$, named $BLT_1$ and $BLT_2$, and at least two distinct receptors for the CysLTs have been isolated and characterized, namely, $CysLT_1$ and $CysLT_2$.

The cloning of BLT receptors in humans[22] and mice[23,24] will help to clarify their signaling pathways and functional roles. Recent studies using $BLT_1$-deficient mice have demonstrated an important role for the $BLT_1$ receptor and $LTB_4$ in the recruitment of T cells to an inflammatory site[25,26,27,28]. In autoimmune inflammatory diseases, the migration of T cells to an inflamed region in the body is essential for a proper response of the adaptive immune system. More specifically, it was demonstrated that the recruitment of a specific population of circulating CD8+ effector T cells depends on selective activation of $BLT_1$ receptors and rapid integrin-mediated cell attachment. In this study, the authors showed that circulating wild-type CD8+ effector T cells more efficiently migrated to the inflamed peritoneal cavity than $BLT_1$ receptor-deficient CD8+ effector T cells[25]. In another study of $BLT_1$ receptor–deficient mice, the investigators obtained evidence for an essential role of $BLT_1$ receptor signaling and CD8+ effector T cells in the development of increased airway hyper-responsiveness (allergic asthma)[28]. These results strongly underline the importance of both $BLT_1$ receptor function and $LTB_4$ with the function of T cells in the etiology of certain chronic inflammatory diseases, knowledge that could be potentially important for targeting individual components of the disease process.

The biological effects of CysLTs are mediated through at least two distinct receptors: $CysLT_1$ and $CysLT_2$. The $CysLT_1$ receptor has a tenfold higher affinity for $LTD_4$ when compared to the low-affinity receptor $CysLT_2$, and with a much higher affinity for $LTD_4$ than $LTC_4$. The $CysLT_2$ receptor, on the other hand, exhibits low, but equal, binding affinities for $LTD_4$ and $LTC_4$. Consequently, the effects of $LTD_4$ are primarily mediated through the $CysLT_1$ receptor, a high-affinity receptor for $LTD_4$ that has been cloned and characterized[29,30].

In addition to the increased downstream signaling activity of $CysLT_1$ receptors in inflammatory and colon cancer tissues, the expression level of the $CysLT_1$ receptor is also increased in colon cancer tissues[19]. In good agreement with this, we also observed an increased expression level of the $CysLT_1$ receptor in different colon cancer cell lines[19]. The increased $CysLT_1$ receptor expression in colon cancer cell lines correlates well with the ability of $LTD_4$ to increase the survival of these cells[20].

Interestingly, in nontransformed cells, we observed that $LTD_4$ stimulation triggered a relocalization of $CysLT_1$ receptors from the plasma membrane to the nucleus and more specifically to the outer nuclear membrane[31]. Even though the functional importance of this translocation is not known yet, we have observed that in human colon cancer specimens and in colon cancer cell lines, the $CysLT_1$ receptor is predominantly localized in the nuclear membrane[31]. In summary, these results strongly suggest that not only up-regulation of $CysLT_1$ receptor expression, but also their localization, have an impact on inflammatory-related development of colon cancer and the functional properties of colon cancer cells.

At present, much less is known about the signaling and functional properties of the $CysLT_2$ receptor. This receptor was originally described in pulmonary vein preparations, but its expression has since been established in a number of other cell types, such as placenta, spleen, heart, leukocytes, brain, and endothelial cells. The expression of the $CysLT_2$ receptor has been shown to be up-regulated in human mast cells in response to the cytokine interleukin (IL)-4[32]. These authors suggested selective functions of the $CysLT_2$ receptor on mast cells, including enhanced generation of IL-8. The enhanced production of IL-8 is interesting since this cytokine is a very potent chemotactic factor for neutrophils, suggesting that $CysLT_2$ receptors on mast cells could be crucial for the recruitment of neutrophils and, thus, for the bronchial response in patients with status asthmatics[32,33].

## INITIAL CYSLT RECEPTOR SIGNALING

Previous observations reported from our laboratory[34] and by other investigators[35] have shown that binding of $LTD_4$ to the $CysLT_1$ receptor results in a downstream signaling cascade that is initiated by an interaction between a heterotrimeric G-protein and a $CysLT_1$ receptor, indicating that the latter is a G-protein coupled receptor (GPCR). On ligand-induced receptor activation, a GPCR is converted from an inactive to an active conformational state in which it can interact and activate heterotrimeric G-proteins.

In general, heterotrimeric G-proteins are classified by reference to their $\alpha$-subunits. These can be divided into four main classes that are designated $G_s$, $G_i$, $G_{q/11}$, and $G_{12\text{-}13}$. In their inactive state, the $\alpha$-subunits of the heterotrimer bind GDP and are associated with a $G\beta\gamma$ dimer. Ligand-induced activation of a GPCR triggers a conformational change of the receptor and an interaction with a heterotrimeric G-protein. This interaction functions as a GDP/GTP exchange factor promoting the release of GDP and the binding of GTP to the $\alpha$-subunit. This, in turn, leads to dissociation between the $\alpha$-subunit and the $G\beta\gamma$-dimer. The released GTP-$G\alpha$ and $G\beta\gamma$ subunits can now interact with a variety of downstream effectors, such as protein kinases, lipases, and ion channels. Activation or inactivation of such effectors will eventually lead to an altered regulation of a number of biological and cellular functions, such as proliferation and survival. Pertussis and cholera toxin are two bacterial toxins that specifically interfere with the activation-inactivation cycle of heterotrimeric G-proteins. Pertussis toxin catalyzes the ADP-ribosylation of the $G\alpha_i$ protein family resulting in an uncoupling of the G-protein from activated GPCRs. Cholera toxin ADP-ribosylates the $G\alpha_s$-subunit, leading to a constitutively active G-protein due to inhibition of the endogenous GTPase activity of the $G\alpha_s$-subunit[36].

One of the hallmarks of $CysLT_1$ receptor signaling is the induction of an intracellular $Ca^{2+}$ signal that originates from an intracellular mobilization and an influx over the plasma membrane. The $CysLT_1$ receptor–induced intracellular mobilization of $Ca^{2+}$ has been shown to be mediated by a pertussis toxin (PTX)–insensitive G-protein, while the influx was mediated by a PTX-sensitive G-protein[37]. The $CysLT_1$ receptor–induced intracellular mobilization of $Ca^{2+}$ in intestinal epithelial cells is mediated via a Rho-dependent activation of phospholipase $C\gamma$[38], while the subsequent influx of $Ca^{2+}$ is regulated by protein kinase A activity at a site downstream of the activation of the PTX-sensitive G-protein in these epithelial cells[39].

It is important to mention that in hematopoetic cells, it appears as if the $CysLT_1$ receptor primarily couples to the heterotrimeric $G_{q/11}$-protein[40]. Activation of the $CysLT_1$ receptor in these cells triggers, as in intestinal epithelial cells, an intracellular $Ca^{2+}$ signal[41]. Furthermore, this $CysLT_1$-induced intracellular $Ca^{2+}$ signal is only partially inhibited by incubation with PTX[41]. The most logical reason for this observation is that $CysLT_1$ also triggers the activation of the PTX-insensitive $G_{q/11}$-protein in these cells[42].

## CYSLT$_1$ RECEPTOR SIGNALING AND CELL SURVIVAL

The pathways leading to apoptosis vary depending on cell type and the situation. In some cases, the process is completely independent of mitochondrial involvement and occurs through a direct sequence of caspase-to-caspase activation. On the other hand, death receptor–mediated pathways, as well as other apoptotic pathways, often proceed through cytochrome c release, via apoptosome assembly, to the activation of downstream executioner caspases.

High levels of LTs and up-regulated expressions of COX-2 and its main product, $PGE_2$, are characteristic hallmarks of inflammation. Moreover, increased expression level and activity of COX-2 have been implicated in the regulation of cell survival and early colon carcinogenesis. As previously mentioned, $LTD_4$ causes an increase in expression and/or nuclear membrane accumulation of COX-2 and, thus, increased production of $PGE_2$ in intestinal epithelial cells. This $LTD_4$-induced COX-2 activation was mediated via the activation of a PTX-sensitive heterotrimeric G-protein and activation of Erk1/2[43]. The effect of $LTD_4$ on PG production can be counteracted by the presence of the COX-2 inhibitor NS-398. If the COX-2 activity was blocked by the presence of NS-398, this also led to a sustained activation of caspase-3 and an increased rate of apoptosis in intestinal epithelial cells, effects that could be reversed by the addition of $LTD_4$[20].

Consequently, in the presence of NS-398, there is an increased apoptotic signaling in intestinal epithelial cells. This signaling cascade includes a caspase 8–dependent cleavage of Bid, resulting in the production of the active form of Bid, tBid (truncated Bid). The newly formed tBid translocates to the mitochondria and induces apoptosis through the release of cytochrome c into the cytoplasm. Release of cytochrome c from the mitochondria in response to NS-398 has also been shown for esophageal and colon cancer cells[44,45]. The ability of $LTD_4$ to reverse the apoptotic effect of NS-398 in intestinal epithelial cells could also be coupled to an up-regulation of Bcl-2 and its accumulation at the mitochondria[46]. Bcl-2 is a well-known protein that functions as a negative regulator of apoptosis. These results indicated that $LTD_4$ could exert its antiapoptotic effect upstream of or at the mitochondrial level. It is still unclear how $LTD_4$ induces translocation of Bcl-2 to the mitochondria, but the Bcl-2–dependent cell survival signaling involved important roles of a $G\alpha_{i3}$-protein, COX-2, and Erk1/2[47].

Apart from Bcl-2, there are a number of other cellular proteins that are intimately connected to the regulation of cell survival and apoptosis; one of these is β-catenin. This protein became very interesting in relation to $LTD_4$-induced survival signaling when we found a novel $LTD_4$-triggered association between Bcl-2 and β-catenin in the mitochondria from intestinal epithelial cell lines[48]. We hypothesize that $LTD_4$ may enhance cell survival via activation and association of β-catenin with Bcl-2 in the mitochondria. These results are in line with recent observations by Shin et al.[49], demonstrating that β-catenin activation and signaling execute an antiapoptotic effect through protection of cytochrome c leakage from the mitochondria.

## CYSLT$_1$ RECEPTOR SIGNALING AFFECTS INTESTINAL CELL ADHESION

It has been known for a long time that adhesion of cells to the extracellular matrix (ECM) is necessary for survival, growth, and migration. Integrins are key mediators of both matrix attachments and signaling responses. Most integrin ligands are ECM proteins, including collagen, laminins, fibronectins, and many other peptides. Individual cells can vary their adhesive properties by selectively enhancing their surface expression of specific integrins during different stages of adhesion and, thus, affect their survival and migration properties.

We have shown that activation of the $CysLT_1$ receptor results in increased adhesion and migration of colon cancer cells on collagen. These responses were shown to be mediated via a $CysLT_1$ receptor activation of COX-2 and a subsequent formation of $PGE_2$ that resulted in an increased expression of α2β1 integrins on the surface of these colon cancer cells[50]. In accordance, it has been demonstrated that inhibition of COX-2 activity in endothelial cells impairs their migration via inhibition of αVβ3 integrin activation in these cells[51]. Consequently, inhibition of COX-2 in colon cancer tissue can have several effects by impairing angiogenesis, as well as tumor cell dissemination and survival. Interestingly, the remodeling of the ECM seen at sites of chronic inflammation[52] involves a thickening of collagen layers observed in patients with different IBD[53]. These changes could possibly contribute to the ability of $CysLT_1$ receptor signaling in intestinal epithelial cells to induce their neoplastic transformation.

Furthermore, $LTD_4$ stimulation of $CysLT_1$ receptors on intestinal epithelial cells also increases integrin-dependent adhesion of these cells to collagen via an increased accumulation of vinculin to the sites of focal adhesion[54]. In addition, the $CysLT_1$ receptor has recently been shown to promote intestinal cell migration via activation of the small GTPase Rac[55]. These results indicate that $CysLT_1$ receptor signaling affects attachment of intestinal epithelial cells to the ECM via several mechanisms, and that this, in turn, constitutes an important regulation of tumor cell survival and migration.

## CYSLT$_1$ RECEPTOR SIGNALING AND CELL PROLIFERATION

The $LTD_4$-induced increase in intestinal epithelial cell number is regulated by dual intracellular signaling pathways: one that is initiated by a PTX-sensitive G-protein and involves activation of Erk1/2 and one that is PTX insensitive, but dependent on Ras activation. The initiation and existence of two parallel pathways is in good agreement with previous reports regarding the $Ca^{2+}$ signaling properties of the $CysLT_1$ receptor[34,37,38]. Intracellular signaling by the $CysLT_1$ receptor can promote an increased number of intestinal epithelial cells by a traditional Ras-dependent pathway. However, more interestingly, even in the absence of a Ras signal, a $G_i$-protein/PKCε/Raf-1/MEK signaling pathway can secure a $CysLT_1$ receptor–induced increase in cell number via activation of Erk1/2, see Fig. 1[43].

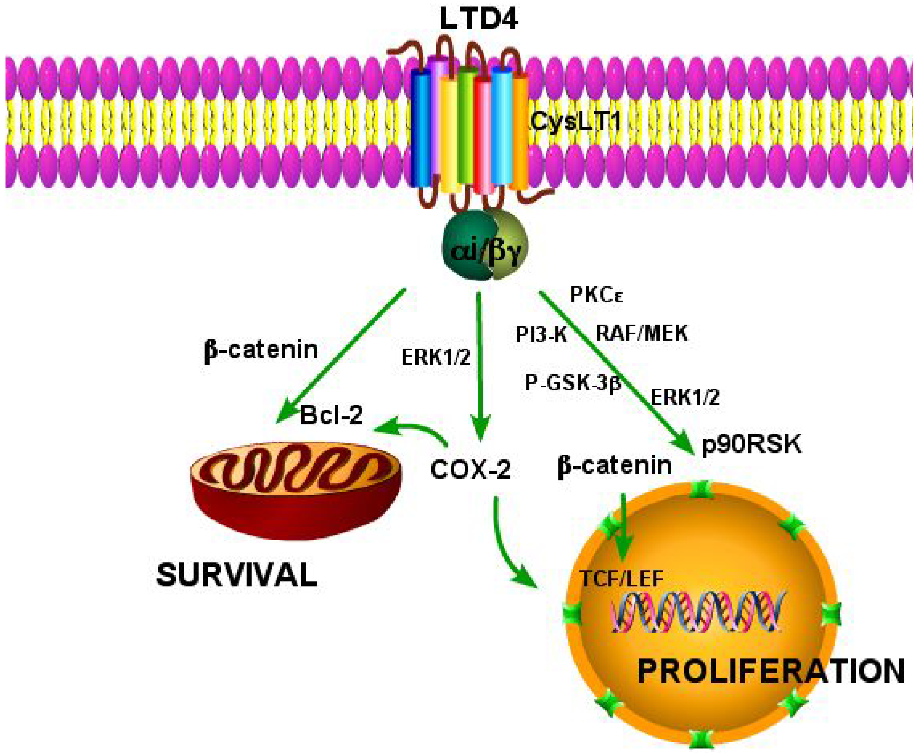

**FIGURE 1.** A simplified signaling cascade leading to $LTD_4$-induced cell survival and cell proliferation.

The $CysLT_1$ receptor–induced increase in intestinal epithelial cell number has been shown to be totally dependent on the activation of the 90-kDa ribosomal S6 kinase (p90RSK) and the cAMP-responsive element-binding protein (CREB)[56]. The $CysLT_1$ receptor–induced Erk1/2-dependent signaling pathway was clearly responsible for the downstream transient and time-dependent activation of p90RSK[56]. p90RSK is a well-known substrate for Erk1/2 and it has been established that both Erk1/2 and p90RSK are translocated to the nucleus where p90RSK can phosphorylate CREB at ser133[57]. In contrast to these results, we could clearly show that the $CysLT_1$ receptor–induced activation of CREB was not mediated via an increased activity of p90RSK. Instead, we found that a $CysLT_1$ receptor–induced activation of protein kinase Cα (PKCα) was responsible for the downstream activation of CREB[56]. Intestinal epithelial cells treated with $LTD_4$ exhibit a significantly reduced G0/G1 phase compared to unstimulated control cells, while inhibition of PKCα or overexpression of kinase dead CREB mutant increased the sub-G0/G1 phase[56]. These data suggest that activation of the $CysLT_1$ receptor regulates proliferation via p90RSK, while it regulates survival via a CREB-dependent pathway.

Activation of such $CysLT_1$ receptor signaling pathways and the subsequent effects on proliferation and survival of intestinal epithelial cells indicate that the inflammatory mediator, $LTD_4$, can contribute to growth of intestinal cells during pathological inflammatory conditions. This, in turn, indicates that the $CysLT_1$ receptor has an important role in neoplastic transformation and development of colon cancer. Furthermore, the recent findings that colon cancer cells exhibit an endogenous production of CysLTs suggest that also after a manifest colon tumor has been established, $CysLT_1$ receptor signaling can mediate important auto- or paracrine effects on the tumor cells and thus promote further tumor growth[58]. Comparatively little is known about the $CysLT_2$ receptor with regard to its intracellular signaling, cellular functions, and role in cancer. The $CysLT_2$ receptor has been detected in neuron- and glial-appearing cells in the area surrounding a tumor. Furthermore, tumors can induce the expression of the $CysLT_2$ receptor in vascular endothelial cells[59]. The expression of the $CysLT_2$ receptor has also been correlated with late astrocytic proliferation following focal cerebral ischemia[60]. Recently, it has been shown that $LTB_4$, via its receptor $BLT_1$, can induce cell proliferation in colon cancer cell lines and that blocking this receptor with a specific receptor antagonist induces apoptosis in colon cancer cells[61]. Indeed, both 5-LO and the $BLT_1$ receptor exhibit increased expression in pancreatic cancer as well as in early pancreatic cancer lesions[62]. Moreover, a phase I clinical trial indicated that LY293111, a well-tolerated orally stable $BLT_1$ receptor antagonist with low side effects, may become a novel drug for treatment of pancreatic cancer. Furthermore, $LTB_4$ has also been reported to play a pivotal role in CD-40–dependent activation of chronic B lymphocytic leukemia cells[63].

In summary, the two enzymes, COX-2 and 5-LO, as well as their metabolites, are not only important mediators of acute and chronic inflammatory conditions, but also have been recognized to be essential regulators of cancer development and progression in several different tumor types[7,8,9,10,11,12,15, 16,17,18,19,58,62,63].

## ACKNOWLEDGMENTS

This work was supported by grants from the Swedish Cancer Foundation, the Swedish Medical Research Council, and the Foundations at Malmö University Hospital.
